# Molecular Mechanisms to Target Cellular Senescence in Aging and Disease

**DOI:** 10.3390/cells11233732

**Published:** 2022-11-23

**Authors:** Serena Marcozzi, Antonio Paolo Beltrami, Marco Malavolta

**Affiliations:** 1 Advanced Technology Center for Aging Research, IRCCS INRCA, 60121 Ancona, Italy; 2 Scientific Direction, IRCCS INRCA, 60124 Ancona, Italy; 3 Institute of Clinical Pathology, Department of Medicine (DAME), University of Udine and Azienda Sanitaria Friuli Centrale (ASUFC), 33100 Udine, Italy

Cellular senescence is a state of irreversible cell cycle arrest in response to several stressors, including DNA damage, increased cellular oxidative stress, telomere shortening, oncogene activation, and a deep epigenetic remodeling [1]. Senescent cells are dynamic and metabolically active cells, characterized by a distinctive secretory phenotype (the so-called senescence associated secretory phenotype, SASP) consisting of bioactive molecules that have deep consequences in surrounding tissues [2]. Among the physiological function of the SASP, it is particularly important that the capacity to attract immune system cells with the function to eliminate the senescent cells and to induce their replacement by healthy cells triggering stem cell plasticity.

In recent decades, the growing interest in this phenomenon, has uncovered several biological implications, both in the context of beneficial as well as deleterious processes. In fact, it is arguable that senescence is a phenomenon positively selected through evolution due to its capability to limit excessive proliferation of cells, promoting, in this way, tissue regeneration, wound healing, and resolution of fibrosis and acting as a barrier against carcinogenesis and viral infections. However, the persistence of senescent cells in various tissues (as observed in aged tissues and age-related diseases), and the consequent continued secretion of SASP factors, results in a chronic pro-inflammatory microenvironment that promotes tissue aging and is involved in the development of cancer, chronic inflammation, immunodeficiency, and stem cell depletion [2]

For this reason, in this Special Issue of *Cells*, the exciting and emerging topic of identifying genetic and pharmacological interventions to eliminate the chronic accumulation of senescent cells, with the intention to prevent chronic diseases and improve health in aging, is addressed in depth (Figure 1a). 

The main emerging field in this research area is the use of senolytic drugs to selectively induce apoptosis in senescent cells. For example, the drug Navitoclax and the peptide FOXO4-DRI, have proven effective in eliminating senescent satellite cells in models of sarcopenia, thus allowing the recovery of the activity of the remaining satellite cells for muscle regeneration [3]. Results along the same line were obtained with the combination of Dasatinib and Quercetin, a widely studied senolytic cocktail, to selectively induce apoptosis in adult cardiac stem/progenitor cells (CSCs) [4]; in addition, the elimination of senescent cells leads to a positive effect on the activation of resident and healthy CSCs, resulting in the formation of new cardiomyocytes, thereby decreasing cardiomyocyte hypertrophy and fibrosis [4,5]. Interestingly, a decrease in the number of senescent cells and a positive effect on proliferation and metabolism of adult CSCs, can be also obtained also when these cells are treated with serum and plasma samples from healthy donors [6]. The p38-MAPK signaling, a cell-specific modulator of proliferation, was additionally identified as the underlying pathways that promote the blood-serum-mediated proliferation of CSCs.

An alternative strategy could be to prevent senescent cells accumulation, for example, by using molecules capable of inhibiting oxidative stress and inflammatory status, such as 3,4-Dihydroxybenzalacetone (DBL), which has shown in vivo cardioprotective potential against age-associated cardiac changes [7], or by using inhibitors of inflammatory mediators that promote cellular senescence, such as visfatin. Visfatin, firstly identified as an adipocytokine released by visceral adipose tissue, was short after reconsidered as a more widely secreted factor and it has been proposed among the factors able to induce senescence in human dental pulp cells [8], as well as in human endothelial cells [9]. In this field of action, another interesting target could be the hypoxia-inducible factor-1α (HIF-1α): several pieces of evidence revealed that the age-dependent impairment of HIF-1α is involved in the development of the senescent phenotype in endothelial cells and in the progression of atherosclerosis. In line with this, HIF-1α overexpression could be a new therapeutical perspective on preventing the development of endothelial cell senescence and all the resulting pathologies [10]

A promising alternative to senescent cells removal by senolytic drugs, are senescence immunotherapy strategies. Indeed, it is emerging that conditions of chronic immunological stress, imposed by chronic inflammation, chronic bacterial infections, or exposure to persistent viral infections, may promote the progression of lymphocytes toward cellular senescence (a factor contributing to the overall process of immunosenescence) [11,12] and impose immune deficits that impair the clearance of senescent cells from tissues, thereby exacerbating the accumulation of senescent cells and their negative implications [13,14]. Valuable tools for senescence immune surveillance may be to engineer T cells or NK cells to express the chimeric antigen receptor (CAR) capable to recognizing ligands on the surface of senescent cells [13]. Additional proposed therapeutic targets are the molecular pathways underlying SASP activation, such as the overexpression of klotho, which has been shown to be effective in preventing the initiation of an immunosenescence-like phenotype in monocytes [15].

The growing understanding of senescence and of the molecular mechanisms to target senescent cells opens the way also for other potential applications. For instance, the induction of senescence is also relevant in cancer therapy: indeed, it is normal to observe an increase in senescent cells in cancer after chemotherapeutic and radiotherapeutic treatment (therapy-induced senescence, TIS), producing a stable cell cycle arrest and, therefore, tumor growth inhibition. Moreover, the derived SASP can reinforce the senescent state in neighboring cancer cells and activate immune cells to recognize and destroy cancer senescent cells, resulting in an overall anti-tumoral effect. On the other hand, an incomplete immune cell-mediated clearance of cancer senescent cells and the consequent long-term state of inflammation caused by the SASP may favor tumor growth and relapse. Therefore, several authors have proposed a “one–two punch” strategy that consists in the combination of senogenic therapies (therapies that induces cellular senescence) and subsequent elimination of senescent cells with senolytic compounds (Figure 1b), which is effective in reducing metastasis and increasing the rate of disease-free survival after chemotherapy [16,17,18]. However, it is critical to consider that not all clinically used antitumoral drugs induce senescence in all cancer cells, underlying a variable susceptibility of cancer cells to undergo senescence upon treatment [19]. In addition, the type of TIS could influence the subsequent senolytic treatment: for example, while the Bcl-2 family anti-apoptotic inhibitor were lethal for prostate cancer cells treated with DNA damage inducers (e.g., irradiation), they were totally ineffective against enzalutamide-TIS cells [20]. Another example of heterogeneous responses has been provided using several cell lines of head and neck squamous cell carcinoma exposed to the same anti-tumor treatment [21]. These results are likely the consequence of the high molecular heterogeneity of this kind of cancer.

Taken together, these results highlight that the increasingly comprehensive understanding of the senescence phenotypes is critical to the design of increasingly fitting interventions for the treatment of age-related chronic disorders and diseases.

## Figures and Tables

**Figure 1:**
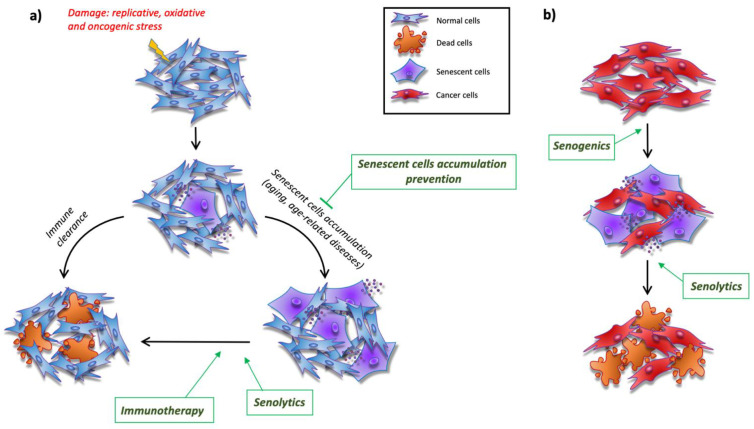
Cartoon summarizing the hypothesized interventions aimed at eradicating the chronic accumulation of senescent cells: (**a**) interventions suggested to prevent chronic diseases and improve health in aging; (**b**) the “one-two punch” strategy combining senogenic and senolytic therapies for the treatment of cancer.
